# Editorial: Causes and consequences of solitude in children and adolescents

**DOI:** 10.3389/fpsyt.2024.1366539

**Published:** 2024-01-26

**Authors:** Xi Chen, Junsheng Liu, Louis A. Schmidt, Xuechen Ding

**Affiliations:** ^1^School of Psychology and Cognitive Science, East China Normal University, Shanghai, China; ^2^Shanghai Key Laboratory of Mental Health and Psychological Crisis Intervention, Shanghai, China; ^3^Shanghai Changning Mental Health Center, Shanghai, China; ^4^Department of Psychology, Neuroscience & Behaviour, McMaster University, Hamilton, ON, Canada; ^5^School of Psychology, Shanghai Normal University, Shanghai, China

**Keywords:** solitude, adjustment, children, adolescents, moderator

Solitude has been conceptualized as *physical* or *perceived* separation from others or a state of *no social interaction* (see McVarnock et al.). Historical perspectives have highlighted both the benefits and costs of solitude for children and adolescents. On the one hand, spending time alone is believed to promote important developmental skills, such as self-regulation and the attainment of autonomy. On the other hand, there is a prevalent concern that excessive time alone will deprive children and adolescents of the valuable and unique opportunities and benefits that come with peer interactions. This paradox illustrates the complex nature of solitude. Nine articles in the Research Topic of “*Causes and Consequences of Solitude in Children and Adolescents*” clarify a broad range of viewpoints and offer substantial empirical evidence to the following themes. The constructs and pathways reviewed or empirically examined in this Research Topic are presented in **Figure 1**.

**Figure 1:**
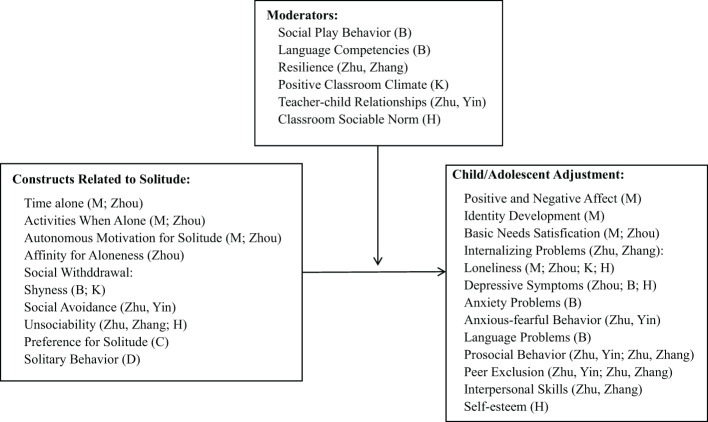
Constructs and pathways reviewed or empirically examined in this Research Topic. M, McVarnock et al.; Zhou, Zhou et al.; B, Baardstu et al.; K, Katulis et al.; Zhu, Yin, Zhu et al.; Zhu, Zhang, Zhu et al.; H, Hu et al.; C, Chen et al.; D , Druskin et al..

## Conceptualization, assessment, and implications of the heterogeneous nature of solitude

McVarnock et al. reviewed the operational definition and measurement of solitude in psychological studies of children, adolescents, and emerging adults since the year 2000. The authors identified 19 empirical studies which measured “spending time alone” using three main approaches: experiments, retrospective reports and experience sampling measures. The majority of these studies focused on merging adults, with a few focused on adolescents and only one examining solitude in children. The considerable variation within measurement approaches, including the operational definitions of “solitude” and the outcomes assessed, may impact study findings. Despite the variation, overall solitude was associated with negative outcomes. However, implications of solitude vary depending on several factors, including activities engaged while alone, and how voluntarily and for what reasons individuals choose to spend time in solitude.

Zhou et al. identified four groups of Chinese late adolescents using latent profile analysis according to the activities they engaged in when alone, their motivation and attitude for solitude and how much time they spent alone. The four groups were: an absence of aloneness group (21.13%), who spent the least time alone; an positive motivational solitude group (29.01%), who reported the highest autonomous motivation for solitude and highest affinity for aloneness; an negative motivational solitude group (38.03%), who reported the highest aversion to aloneness; and an activity-oriented solitude group, who reported most likely to engage in physical activities when alone. Among the four groups, the negative motivational solitude group reported the highest levels of loneliness and depressive symptoms and the lowest levels of basic needs satisfaction; the absence of aloneness group reported the lowest levels of loneliness; the other two groups fell in between. This study sheds light on the heterogeneous nature of solitude. Echoing McVarnock et al., the findings highlight the importance to consider activities while alone and motivations for solitude when examining implications of solitude for adjustment.

## The complex relations between social withdrawal and adjustment in children and adolescents

Social withdrawal, i.e., children choosing to reduce engagement in peer interaction, is one of the major causes of solitude (see McVarnock et al.). There are three types of social withdrawal, characterized by specific combinations of social approach and avoidance motivations: shyness (high approach; high avoidance), social avoidance (low approach; high avoidance) and unsociability (low approach; low avoidance). Overall, social withdrawal is associated with maladjustment, and several articles examined the moderating effects of individual and environmental factors on this association.

Baardstu et al. found that childhood shyness from 18 months to five years old predicted internalizing difficulties and language problems at eight years old. In addition, high levels of language competencies and social play behaviors buffered against later anxiety problems among shy children. Katulis et al. found that shyness, emotional reactivity, and rejection sensitivity in grades 5-7 predicted loneliness 4-5 months later, and these associations were mitigated by positive classroom climate.

Zhu et al. found that higher social avoidance was related to higher peer exclusion and lower prosocial behavior among Chinese migrant preschoolers. In addition, teacher-child relationship moderated these associations. Specifically, the association between social avoidance and peer exclusion was weaker for children with higher teacher-child closeness, whereas the associations between social avoidance and peer exclusion and anxious-fearful behavior were stronger for children with higher teacher-child conflict.

Zhu et al. found that higher unsociability was related with higher peer exclusion and internalizing problems, and related with lower prosocial behaviors and interpersonal skills among Chinese migrant preschoolers. Moreover, these associations were buffered by children’s resilience. Hu et al. found that higher unsociability was related with higher depression and loneliness, and related with lower self-esteem among Chinese adolescents in grades 4-8. In addition, these relations were exacerbated in classrooms with high sociable norm.

## Changes in solitude motivation from a developmental perspective

During the transition from childhood to adolescence, individuals’ preference for solitude (PFS) increases as they grow older. Adolescents need to find a balance between the desire for social connection and the desire for independence. Because these desires are shaped by the social-cultural contexts, the rate at which PFS develops may vary across social-cultural contexts. Chen et al. found that for both urban and rural Chinese adolescent, PFS increased from Grade 6 to Grade 8. Moreover, in accordance with the more salient individualistic values in urban regions, PFS increased faster among urban adolescents than rural adolescents.

Besides the articles mentioned above, Druskin et al. observed preschoolers’ in-school social behaviors and found that compared with typically developing children, children with high behavioral inhibition showed more reticent and solitary behavior, and less social play and teacher interaction.

In summary, articles in this Research Topic include diverse samples and cover a wide age range from preschool years to late adolescence. Several articles advance our understanding about heterogeneity of solitude and its development. Findings of the articles, especially those regarding moderators between social withdrawal and children’s adjustment, have important practical implications for reducing adverse implications of solitude for child and adolescent development.

## Author contributions

XC: Writing – original draft, Writing – review & editing. JL: Writing – review & editing. LS: Writing – review & editing. XD: Writing – review & editing.

